# The effectiveness of nudge-based physical activity interventions in frail older adults: a systematic review

**DOI:** 10.1186/s12966-026-01948-3

**Published:** 2026-07-07

**Authors:** Yang Li, Xiaoxing Lai, Yutong Cui, Xiaopeng Huo, Donglei Shi, Yonghua Cai, Ping Jiang, Shuai Zhang

**Affiliations:** 1 Department of Neurology, Peking Union Medical College Hospital, Chinese Academy of Medical Sciences & Peking Union Medical College, Beijing, 100730 China; 2 Department of Nursing, Peking Union Medical College Hospital, Chinese Academy of Medical Sciences & Peking Union Medical College, Beijing, 100730 China

**Keywords:** Nudge, Frailty, Physical activity, Older adults, Systematic review, Meta-analysis

## Abstract

**Background:**

Frailty is a prevalent geriatric syndrome in older adults, characterized by reduced physiological reserves and increased vulnerability to adverse health outcomes. Physical inactivity is a key modifiable risk factor that accelerates frailty progression. Nudge-based interventions, which subtly guide behavior through choice architecture, offer a promising, low-cost approach to promoting physical activity and improving health outcomes in this population

**Methods:**

A systematic search was conducted in PubMed, EMBASE, Cochrane Library, Web of Science, PsycINFO, and EconLit from inception to May 20, 2025, for randomized controlled trials (RCTs) on nudge-based physical activity interventions in frail older adults. Two reviewers independently performed study selection, data extraction, and quality assessment using the Cochrane risk-of-bias tool. Pooled effect sizes were calculated using random- or fixed-effects models as appropriate, with standardized mean difference (SMD) or mean difference (MD) reported for continuous outcomes. Statistical heterogeneity was assessed using I² and Cochran’s Q tests.

**Results:**

A total of 16 studies involving 2,730 participants were included. Meta-analysis indicated that physical activity interventions based on nudge strategies significantly improved depressive symptoms [SMD = − 0.30, 95% CI (− 0.51, − 0.09), P = 0.016], enhanced cognitive function [SMD = 0.47, 95% CI (0.26, 0.69), P = 0.005], increased physical activity levels [SMD = 1.09, 95% CI (0.43, 1.75), P = 0.001], improved gait speed [MD = 0.07, 95% CI (0.03, 0.11), P = 0.001], enhanced quality of life (QoL) [SMD = 1.02, 95% CI (0.09, 1.94), P = 0.031], and reduced frailty scores [SMD = − 2.00, 95% CI (− 3.01, − 1.00), P < 0.001]. Although improvements were observed in self-efficacy, grip strength, balance ability, and Timed Up and Go (TUG) test performance, these differences were not statistically significant (P > 0.05).

**Conclusions:**

Physical activity interventions based on nudge strategies can significantly increase physical activity levels, effectively improve frailty status, depressive symptoms, cognitive function, gait speed, and QoL among frail older adults. These interventions represent a low-cost and highly feasible behavioral approach for promoting healthy aging.

**Registration:**

CRD420251229406

**Supplementary Information:**

The online version contains supplementary material available at https://doi.org/10.1186/s12966-026-01948-3.

## Background

With the rapid acceleration of global population aging, frailty has emerged as a major and increasingly prominent public health concern. Frailty is a clinical syndrome characterized by age-related decline in physiological reserves and multisystem dysregulation, which compromises the body’s ability to respond to stressors and markedly increases the risk of adverse health outcomes such as falls, disability, hospitalization, and mortality [1–3]. Evidence suggests that insufficient physical activity is an important and modifiable factor accelerating the progression of frailty in older adults. Among individuals with cognitive frailty, active engagement in physical activity has been associated with a 36% reduction in mortality risk [4]. Physical activity behavior is governed by both rational and intuitive cognitive processes [5]. Similarly, *nudge strategies*—rooted in the dual-process theory of decision-making—aim to subtly influence individuals’ automatic or unconscious decisions by optimizing the choice architecture, thereby improving adherence to healthy behaviors without restricting personal autonomy [6]. Münscher [7] categorized nudge strategies into three main types based on the choice architecture framework: (1) modifying the presentation of decision information, (2) optimizing the structure of decision environments, and (3) providing decision support tools. For the purposes of this systematic review, we adopted an operational definition of nudge-based physical activity interventions derived from Thaler and Sunstein’s classic formulation [8] and Landais et al. [9] extended conceptualization. Specifically, an intervention was considered nudge-based if it combined physical activity/exercise content with choice architecture modifications meeting three core criteria: (1) the intervention involves choice architecture design, defined as modifying the physical, social, or informational environment in which decisions about physical activity are made, (2) the intervention does not restrict freedom of choice or forbid any options (i.e., it is non-coercive and easy to avoid), and (3) the intervention does not significantly change economic incentives (i.e., no financial rewards or penalties) [8, 10]. In recent years, several studies have demonstrated the feasibility and preliminary effectiveness of using nudge strategies to enhance physical activity levels among frail older adults [11–12]. However, these studies vary considerably in intervention design, nudge types, implementation contexts, and outcome measures, leading to inconsistent overall findings. Moreover, existing evidence primarily focuses on changes in physical activity per se, with limited systematic evaluation of broader multidimensional health outcomes such as frailty status, physical function, psychological well-being, cognitive function, and quality of life (QoL). Therefore, this study aimed to comprehensively assess, through systematic review and meta-analysis, the overall health benefits of physical activity interventions incorporating nudge strategies among frail older adults. The findings are expected to provide a more scientific and targeted basis for managing physical activity and improving the health and QoL of frail older adults.

## Methods

### Search strategy

This systematic review was conducted in accordance with the PRISMA (Preferred Reporting Items for Systematic Reviews and Meta-Analyses) guidelines. A comprehensive search was performed across nine major databases, including PubMed, EMBASE, Cochrane Library, Web of Science, PsycINFO, EconLit, VIP, Wanfang, and CNKI, covering the period from database inception to May 20, 2025. Based on the PICO principle, the search terms were constructed around four core concepts: physical activity, older adults, frailty, and nudge. A combined strategy of free-text terms and controlled vocabulary (such as MeSH terms in PubMed) was applied. The search strategy was developed using Boolean operators to combine the components as follows: *(physical activity terms) AND (frailty terms) AND (older adult terms) AND (nudge strategy terms).* The detailed search strategy, using PubMed as an example (Supplementary file 1, Table S1). All searches were conducted independently by two researchers, and any discrepancies were resolved through consultation with a third party.

### Inclusion and exclusion criteria

Inclusion criteria: The inclusion criteria were designed according to the PICO framework: (1) Participants (P): Frail older adults aged ≥ 65 years; (2) Intervention (I): The intervention group received an intervention incorporating physical activity or exercise content that included at least one nudge strategy, Consistent with the inclusive approach used in recent systematic reviews of nudge-based health interventions [13], multicomponent interventions combining nudge strategies with non-nudge elements (e.g., health education, structured exercise, or professional guidance) were eligible for inclusion, provided that the nudge component was explicitly described and served as the primary distinguishing feature between the intervention and control groups; (3) Comparison (C): The control group received routine interventions that did not involve nudge strategies; (4) Outcomes (O): Indicators related to physical activity, as well as psychological, cognitive, behavioral, and functional outcomes. Exclusion criteria: (1) Animal experiments; (2) Reviews, dissertations, or case reports; (3) Studies for which valid data could not be obtained; (4) Duplicate publications.

### Literature screening and data extraction

Two researchers independently screened the titles and abstracts, reviewed full texts, extracted data, and assessed the quality of included studies according to the inclusion and exclusion criteria. For nudge identification and classification, two reviewers independently parsed each intervention into discrete behavior-change components and classified them as nudge or non-nudge elements. Specifically, Nudge strategies were categorized into three domains: (A) denotes interventions involving modification of decision information (Translate information, Includes: reframe, simplify; Make information visible, Includes: feedback, make external information visible; Provide social reference point, Includes: refer to descriptive norm, refer to opinion leader.); (B) denotes interventions involving modification of decision structure (Change choice defaults, Includes: set no-action default, use prompted choice; Change option-related effort, Includes: increase/decrease physical/financial effort; Change range or composition of options, Includes: change categories, change grouping of options; Change option consequences, Includes: connect decision to benefit/cost, change social consequences of the decision.); (C) denotes interventions providing decision assistance (Provide reminders; Facilitate commitment, Includes: support self-commitment/public commitment). An intervention was classified as nudge-based if the intervention group incorporated at least one nudge strategy that served as the distinguishing component—that is, a choice architecture element present in the intervention group but absent from the control group—allowing the between-group effect to be attributed to the nudge. Each study was coded for the specific nudge domain(s) applied (A, B, C, or combinations. Any disagreements were resolved through discussion or adjudication by a third party. The following data were extracted: first author (year), country/region, sample size, (intervention/control), age (mean ± SD), nudge strategy type(s) (A/B/C), distinguishing intervention components relative to the control group, intervention duration, assessment time points, and outcome indicators.

### Quality assessment

The quality of the included studies was evaluated using the Cochrane Risk-of-Bias Assessment Tool, which considers seven domains: (1) random sequence generation; (2) allocation concealment; (3) blinding of participants and personnel; (4) blinding of outcome assessors; (5) incomplete outcome data; (6) selective outcome reporting; (7) other potential sources of bias. Each domain was classified as “low risk,” “unclear risk,” or “high risk” according to predefined criteria.

Overall risk-of-bias was judged as low if all four key domains (random sequence generation, allocation concealment, blinding of participants and researchers, and blinding of outcome assessors) were rated as low risk; unclear if any key domain was rated as unclear risk in the absence of any high-risk rating; and high if any key domain was rated as high risk.

### Statistical analysis

Meta-analysis was performed using *Stata* version 14.0. For continuous variables, the mean and standard deviation of post-intervention outcomes were extracted from each study. For dichotomous variables, the number of events and total sample size in each group were recorded. Statistical heterogeneity among studies was assessed using Cochran’s Q test and the I² statistic. An *I²* value < 50% indicated low to moderate heterogeneity, whereas *I²* ≥50% indicated substantial heterogeneity [14]. For effect size estimation: When continuous variables were measured in the same unit, the Mean Difference (MD) was used; When different measurement units were used, the Standardized Mean Difference (SMD) was applied. All meta-analyses were conducted using a random-effects model with the DerSimonian-Laird (DL) estimator, which allows for between-study variability and provides more conservative estimates. Leave-one-out sensitivity analysis was performed by iteratively omitting each individual study and re-estimating the pooled effect to evaluate the robustness of the overall findings and the influence of single studies on the combined results. Funnel plots were generated for the primary outcomes, and potential publication bias was quantitatively assessed using Begg’s rank correlation test and Egger’s linear regression test. A two-sided α = 0.05 was considered statistically significant (*P* < 0.05).

## Results

### Literature screening results

**Fig. 1:**
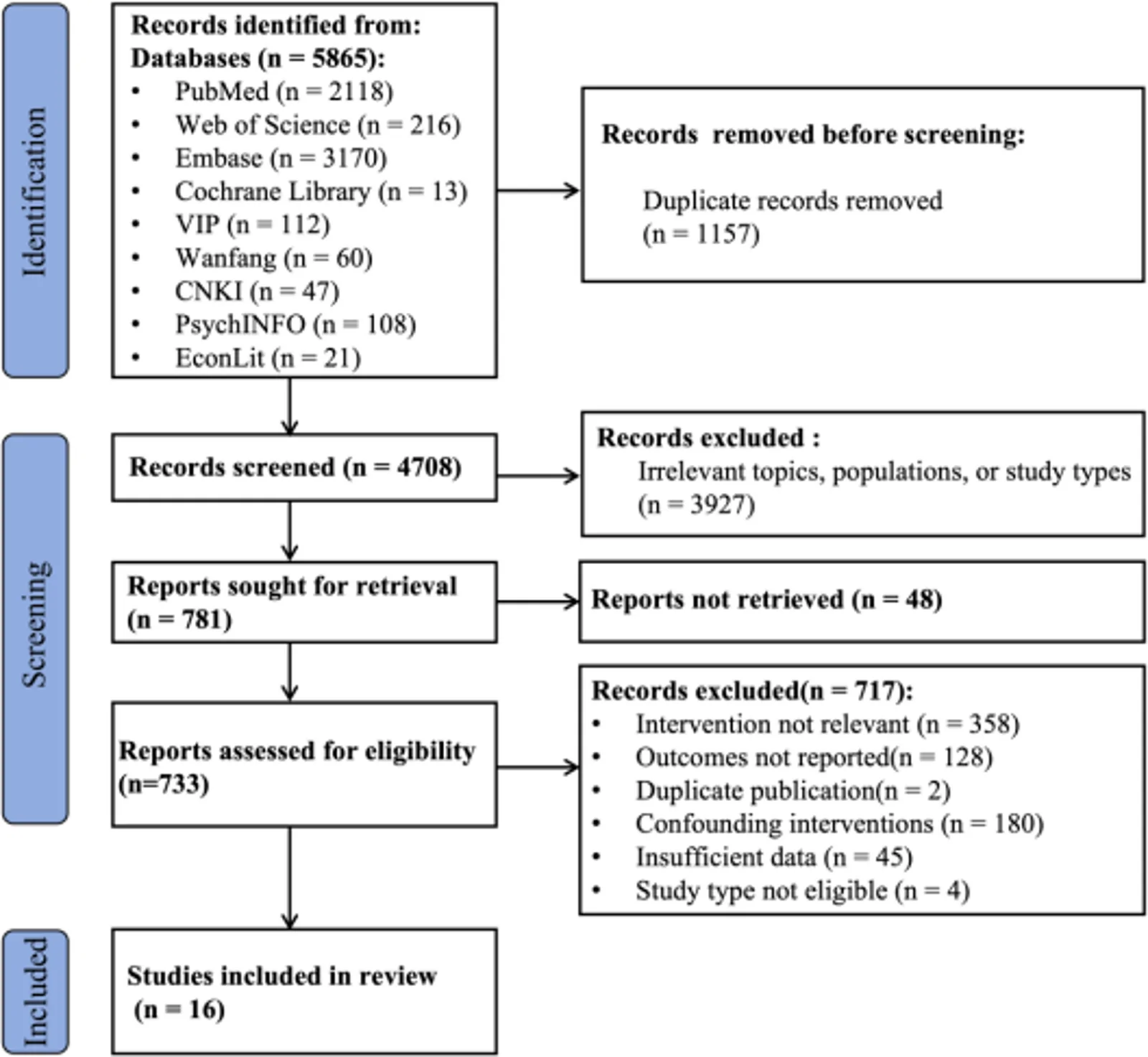
PRISMA flow chart of study selection

A total of 5,865 studies were initially identified. After removing duplicates, 4,708 studies remained. Following title and abstract screening, 3,927 studies were excluded. Of the records undergoing full-text review, 48 could not be retrieved. These included older publications with discontinued electronic access, index-only database entries, and non-journal literature unavailable in full text through any of the searched databases or open - access platforms. Retrieval attempts via interlibrary services and direct author contact were unsuccessful. Ultimately, 16 studies were included after full - text review [15–30]. The literature screening process is illustrated in Fig. 1.

### Basic characteristics of the included studies

The included studies were published between 2010 and 2025, involving a total of 2,730 older adults—1,402 in the intervention groups and 1,328 in the control groups. The basic characteristics of the included studies are summarized in Table 1.

**Table 1:** Basic Characteristics of the included studies

Included Study	Country/Region	Sample Size (I/C) (*n*)	Age (years, $\overline{x}$ ± s)	Intervention Measures			Evaluation Time	Relevant Outcome Indicators
				Nudge Strategy	Distinctive Intervention Content Compared with Control Group	Intervention Duration		
Apóstolo et al. (2019) [26]	Portugal	23/21	80.54 ± 8.47	A, B, C	Cognitive training courses, group training and peer support, animal-assisted activities, professional guidance, reminders	12 weeks	②	Psychological indicators, cognitive indicators, and physical function indicators
Chen et al. (2020) [25]	Taiwan, China	549/533	75.90 ± 7.10	A, B, C	Health education, group training, competitions, reminders, pedometer distribution for monitoring, community leader involvement, motivation, multidisciplinary support	48 weeks	②③	Physical activity status, psychological indicators, physical function indicators, and frailty indicators
de Vries et al. (2016) [27]	Netherlands	64/65	78.40 ± 5.50	A, B, C	Health education and feedback, personal goal setting, goal orientation, self-management guidance and motivation, family and social support	24 weeks	①②	Physical activity status, physical function indicators, frailty indicators, and quality of life
Fang et al. (2024) [21]	China	72/72	73.96 ± 7.20	A, B, C	Health education, feedback, multicomponent exercise design, community supervision, dynamic intensity adjustment, group-based training, empowerment education	24 weeks	①②	Psychological indicators and quality of life
González-Mariscal et al. (2025) [17]	Spain	109/90	74.2 ± 6.40	A, B, C	Providing health information, group discussions for experience sharing, feedback and encouragement, activities in convenient community locations, problem-solving and support, providing guidelines and tools	24 weeks	②③	Frailty indicators
Ha et al. (2020) [15]	South Korea	20/20	77.10 ± 6.40	A, B, C	Structured exercise guidelines, health education, behavioral feedback, personal goal setting, dynamic intensity adjustment, reminders, encouragement	12 weeks	①②	Physical activity status, psychological indicators, frailty indicators, and physical function indicators
Hagedorn et al.(2010) [30]	Denmark	15/12	81.50 ± 7.70	B, C	Visual feedback, gamified exercise, adaptive difficulty adjustment, competition and rewards, feedback to reinforce positive behavior	12 weeks	②	Physical function indicators and psychological indicators
Liu et al. (2017) [19]	Hong Kong, China	58/21	79.32 ± 7.72	A, B, C	Goal visualization, feedback and encouragement, CD and manual for home exercise guidance, coping strategies for barriers, peer experience sharing, self-efficacy enhancement	16 weeks	②	Psychological indicators and physical function indicators
Liu et al. (2021) [29]	Hong Kong, China	22/18	72.10 ± 3.70	A, B, C	Provision of wearable devices, group training, dynamic intensity adjustment, music accompaniment and gamified elements to enhance engagement, consumable materials distribution, feedback and encouragement, peer communication	14 weeks	②③	Physical activity status, psychological indicators, physical function indicators, and frailty indicators
Luger et al. (2016) [18]	Austria	40/40	82.80 ± 8.00	A, B, C	Health education, providing normative information, personal goal setting, social support	12 weeks	②	Frailty indicators
Romera-Liebana et al. (2018) [23]	Spain	176/176	77.20 ± 6.80	A, B, C	Health education, multicomponent exercise design, group activities, dynamic intensity adjustment, professional guidance and management	12 weeks	②③	Physical function indicators
Song et al. (2022) [28]	South Korea	62/64	78.79 ± 5.39	A, B, C	Group training, exercise manuals, enhancing activity enjoyment, health education, motivation, reminders, environmental optimization	12 weeks	②③	Physical activity status, psychological indicators, physical function indicators, and frailty indicators
Tou et al. (2021) [24]	Singapore	30/31	72.07 ± 8.14	A, B, C	Health lectures, structured exercise programs, group activities and peer motivation, professional guidance, feedback	12 weeks	②	Physical function indicators and frailty indicators
Tuan et al. (2024) [22]	Taiwan, China	30/30	78.83 ± 7.70	B, C	Gamified exercise, goal and sensory feedback, dynamic intensity adjustment, safety adaptation, professional supervision and support, motivation, environmental optimization	12 weeks	①②	Physical function indicators, cognitive function, and quality of life
Wong et al. (2024) [20]	Hong Kong, China	101/101	78.80 ± 7.80	B, C	Personalized exercise program, gamified exercise, encouragement, intergenerational support	18 weeks	②	Physical activity status, psychological indicators, cognitive indicators, physical function indicators, and quality of life
Xue et al. (2024) [16]	China	31/34	73.65 ± 3.99	A, B, C	Application training video display, WeChat health knowledge messages, graded exercise design, reminders, feedback, peer support, exercise completion rewards	12 weeks	②③	Cognitive indicators, physical function indicators, frailty indicators, and psychological indicators

I represents the intervention group, C represents the control group

A denotes interventions involving modification of decision information, B denotes interventions involving modification of decision structure, C denotes interventions providing decision assistance;

① indicates outcomes measured during the intervention, ② indicates post-intervention outcomes, ③ indicates follow-up outcomes

### Quality assessment of the included studies

**Fig. 2:**
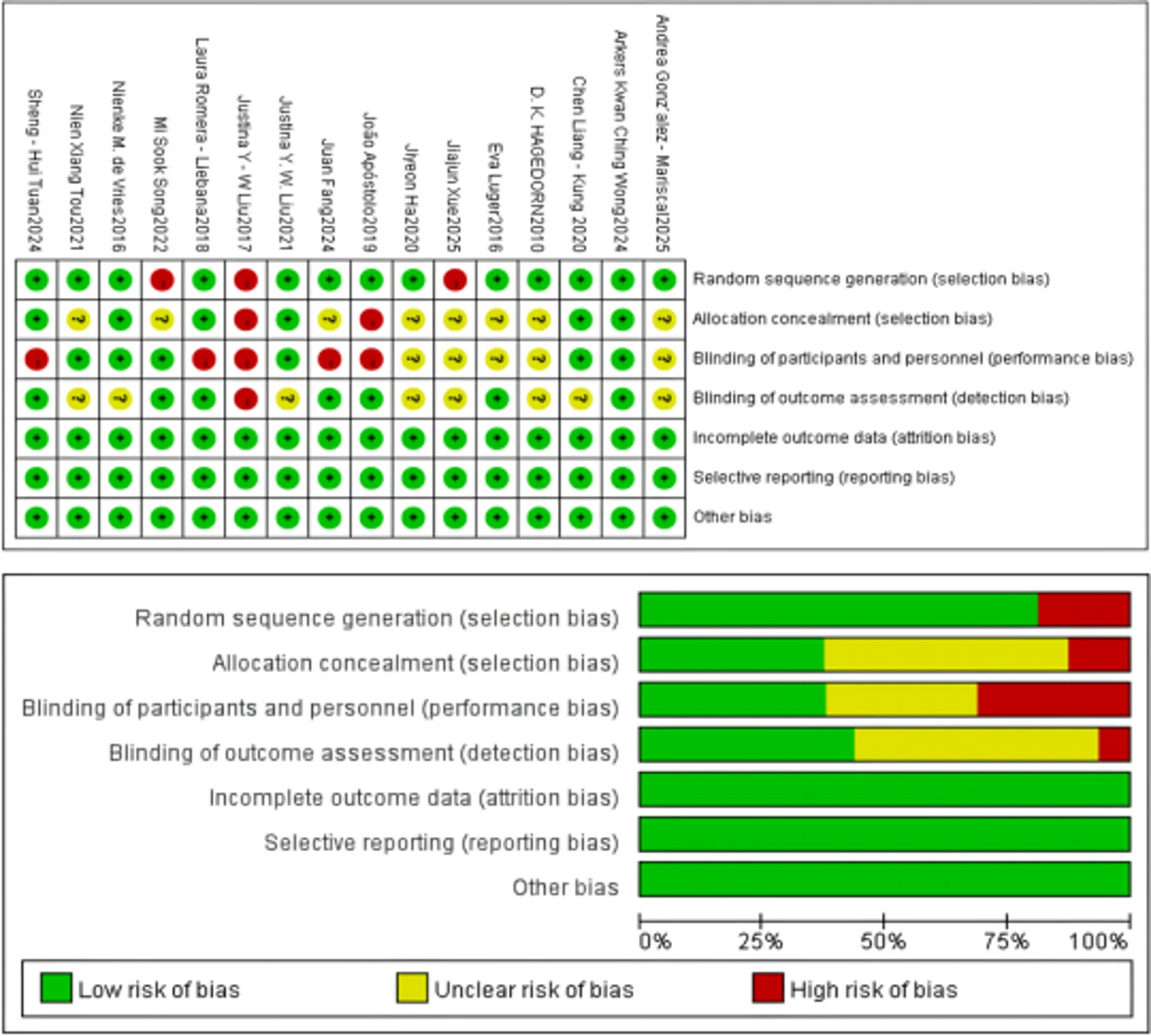
Risk-of-bias assessment of included studies

The methodological quality of the 16 included studies was evaluated using the Cochrane Risk-of-Bias Tool. Among the included studies, 18.8% exhibited a high risk-of-bias in random sequence generation, while 25% showed a high risk-of-bias in allocation concealment. Several studies did not clearly describe their randomization or allocation methods, introducing uncertainty regarding methodological rigor. Regarding blinding, around 50% of the studies were judged to have an unclear or high risk-of-bias in the blinding of participants and personnel. However, all studies were assessed as having a low risk-of-bias for data completeness, selective reporting, and other potential sources of bias, indicating relatively standardized data handling and reporting. The detailed risk-of-bias assessment results are presented in Fig. 2.

### Meta-analysis results

#### Physical activity

**Fig. 3:**
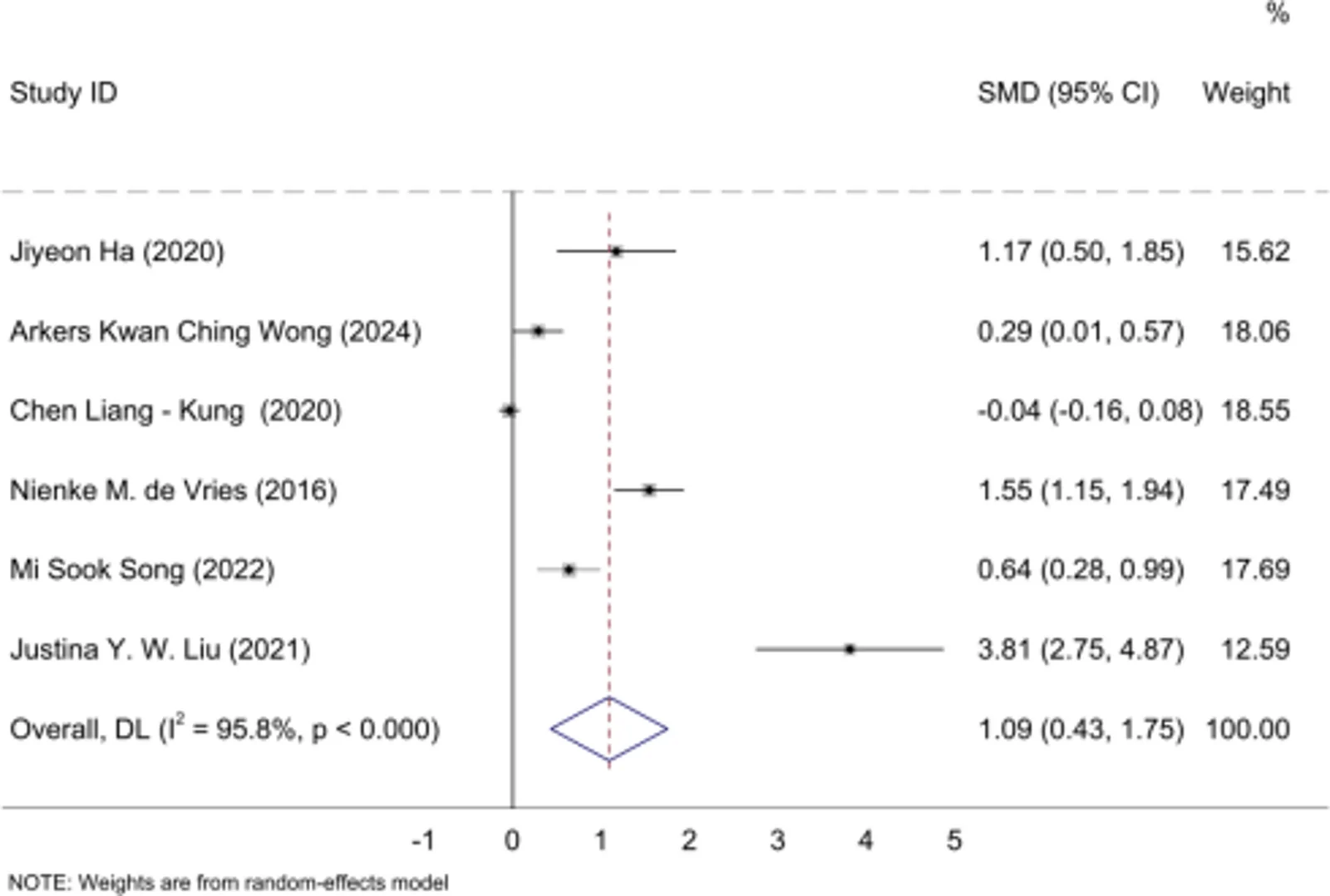
Forest plot of physical activity outcomes

A total of six studies [15, 20, 25, 27–29] were included in the meta-analysis assessing physical activity outcomes. Across these studies, physical activity was measured predominantly through self-report questionnaires assessing activity frequency, duration, and intensity; one study additionally employed objective device-based monitoring (accelerometry and wearable activity trackers). Tests for heterogeneity indicated a high level of statistical heterogeneity among studies (I² = 95.8%, *P* < 0.001). The results showed that post-intervention physical activity levels were significantly higher in the nudge-based intervention group compared with the control group, and the difference was statistically significant [SMD = 1.09, 95% CI (0.43, 1.75), *P* = 0.001], as shown in Fig. 3.

### Frailty and physical function–related indicators

#### Frailty status

**Fig. 4:**
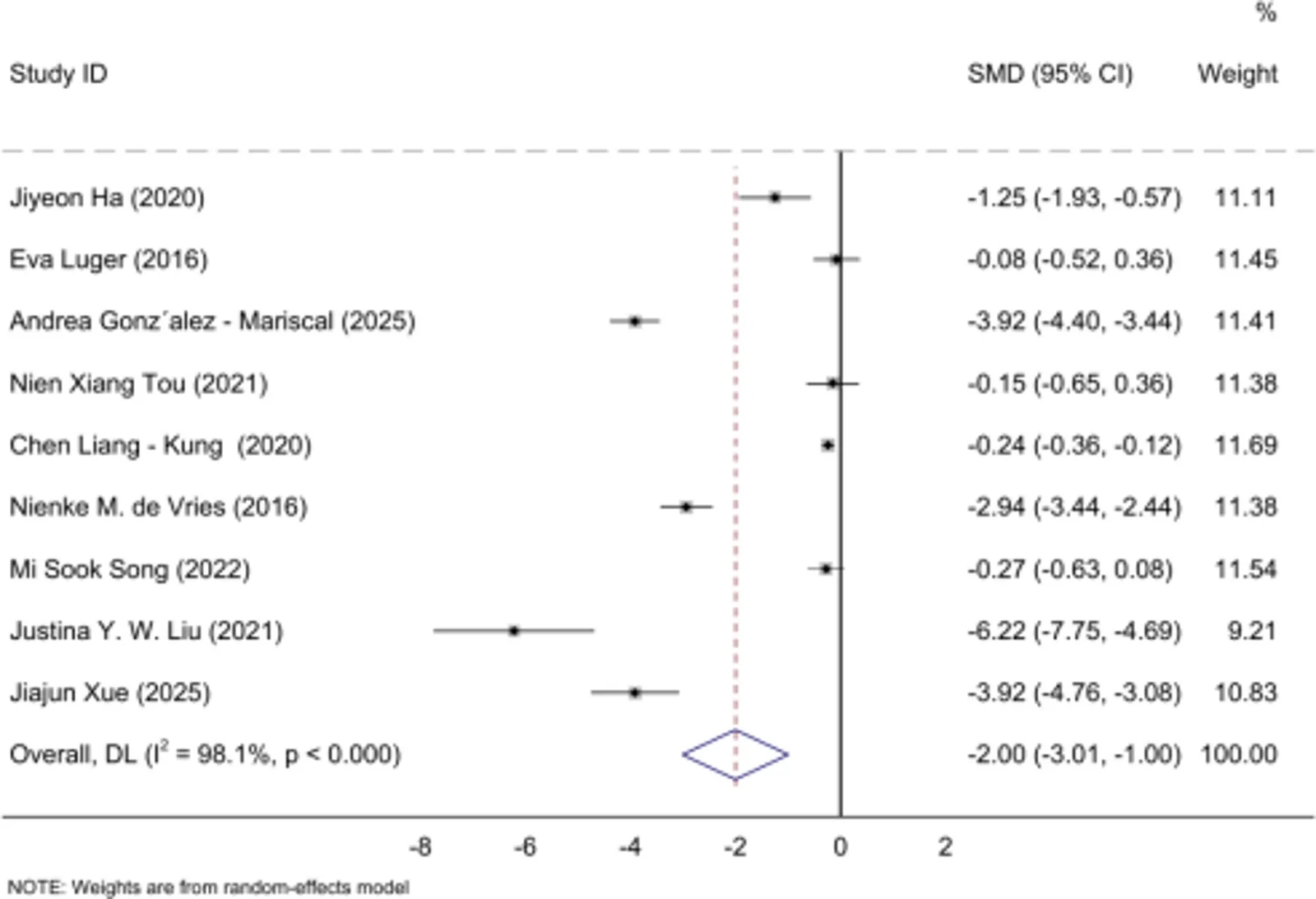
Forest plot of frailty score analysis

A total of nine studies were included in the analysis of the effects of physical activity–based nudging interventions on frailty improvement^[15–18,24−25,27–29]^. Frailty was predominantly assessed using the Fried frailty phenotype, with a minority of studies employing the Survey of Health, Ageing and Retirement in Europe Frailty Instrument (SHARE-FI) or composite frailty indices. The heterogeneity test indicated substantial heterogeneity among studies (I² = 98.1%, *P* < 0.001). The meta-analysis revealed that post-intervention frailty scores were significantly lower in the intervention group compared with the control group [SMD = − 2.00, 95% CI (–3.01, − 1.00), *P* < 0.001] (Fig. 4).

### Balance ability

**Fig. 5:**
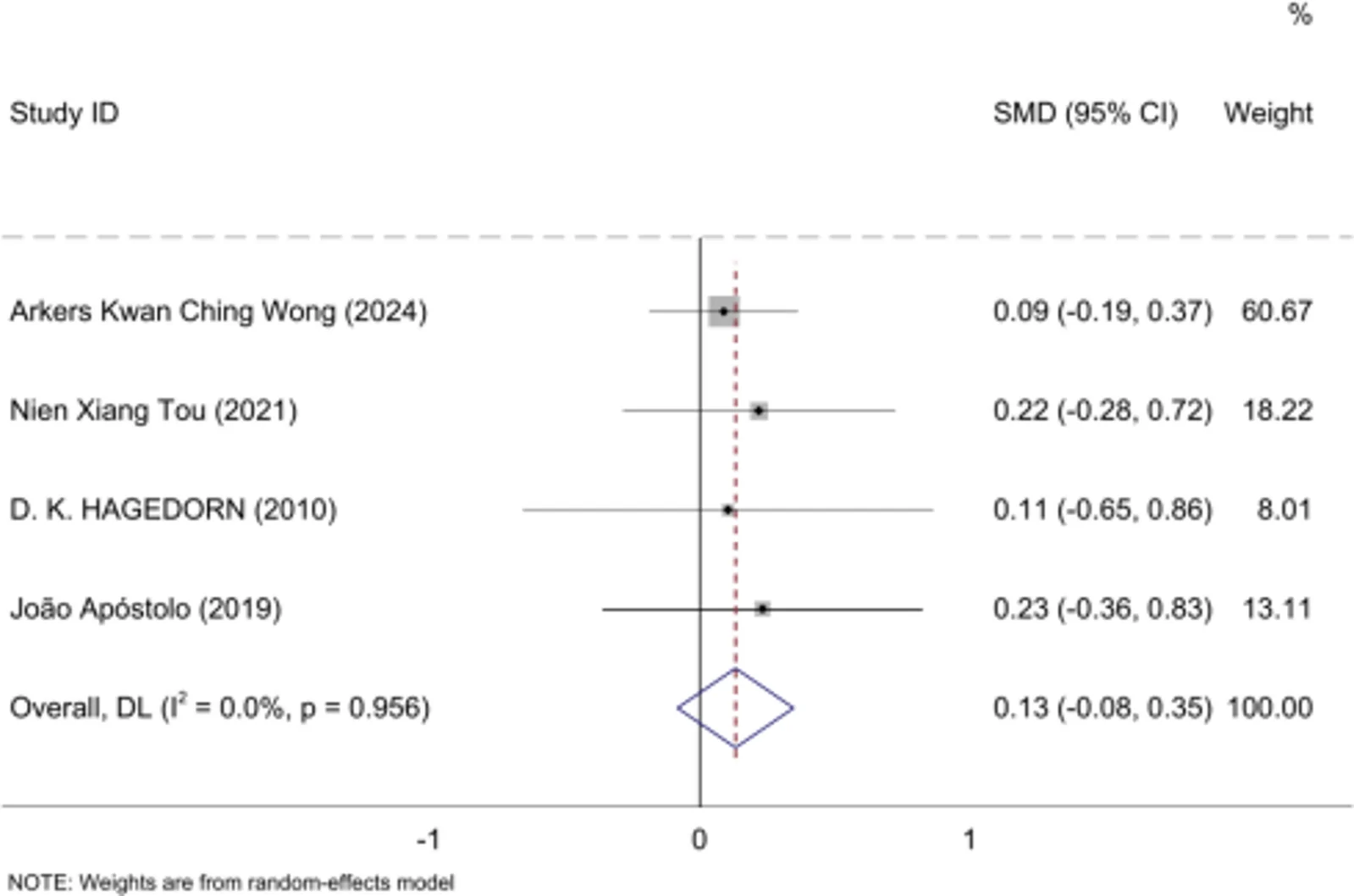
Forest plot of balance ability analysis

Four studies were included in the analysis of balance ability [20, 24, 26, 30]. Balance ability was assessed using the Berg Balance Scale, the balance subscale of the Short Physical Performance Battery, the Tinetti Index, and the Modified Clinical Test of Sensory Interaction and Balance. The heterogeneity test indicated no heterogeneity among studies (I² = 0.0%, *P* = 0.956). The results showed that post-intervention balance ability scores were slightly higher in the nudging intervention group than in the control group, although the effect did not reach statistical significance [SMD = 0.13, 95% CI (–0.08, 0.35), *P* = 0.225] **(**Fig. 5**)**.

### Grip strength

**Fig. 6:**
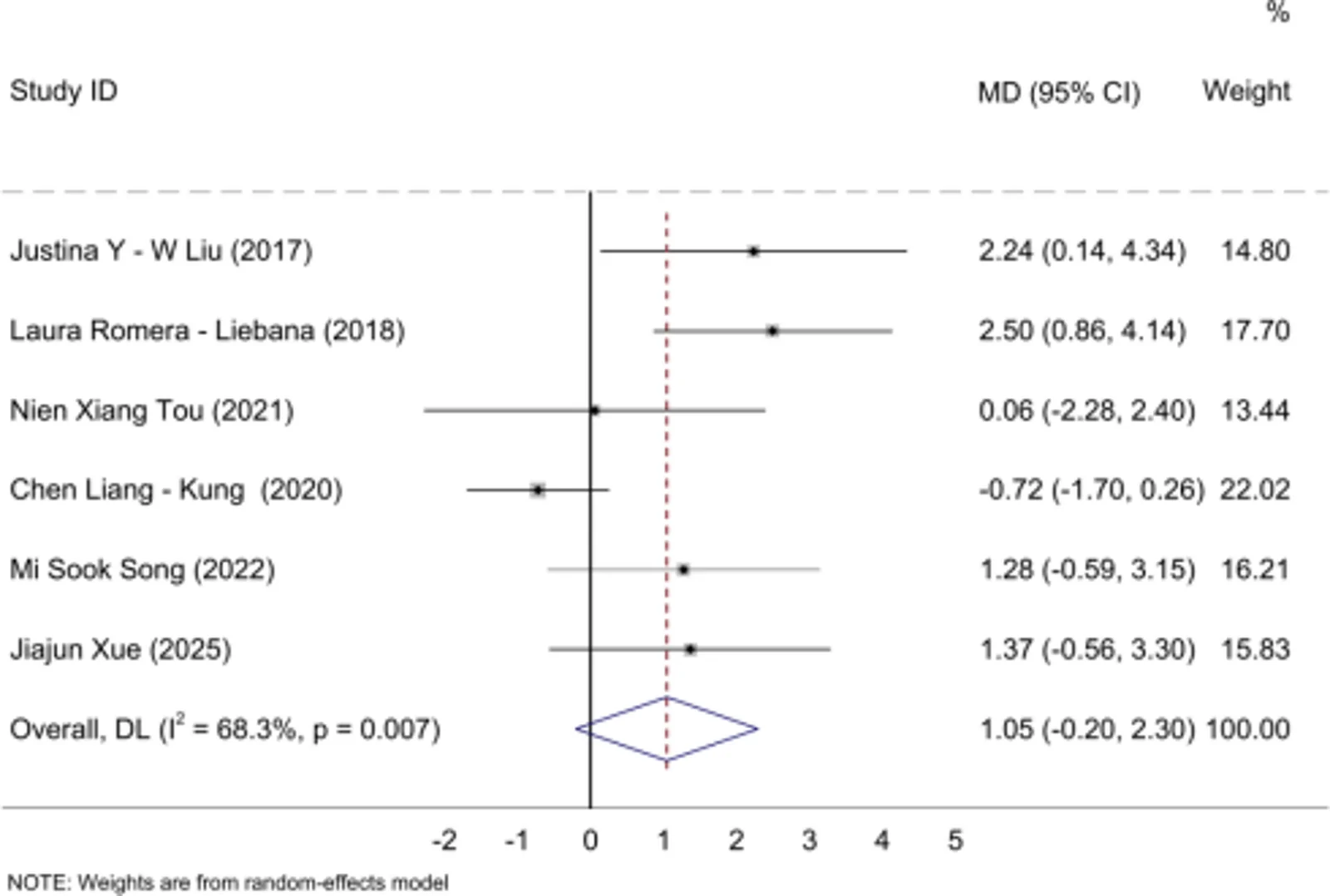
Forest plot of grip strength (kg) analysis

Six studies were included in the analysis of grip strength [16, 19, 23–25, 28]. Grip strength was assessed using handheld dynamometry (most commonly Jamar devices), with values recorded in kilograms as the best or average of multiple trials. The heterogeneity test indicated considerable heterogeneity among studies (I² = 68.3%, *P* = 0.007). The results demonstrated modestly higher post-intervention grip strength in the nudging intervention group compared with the control group, though the difference was not statistically significant [MD = 1.05, 95% CI (–0.20, 2.30), *P* = 0.100] (Fig. 6).

### Gait speed

**Fig. 7:**
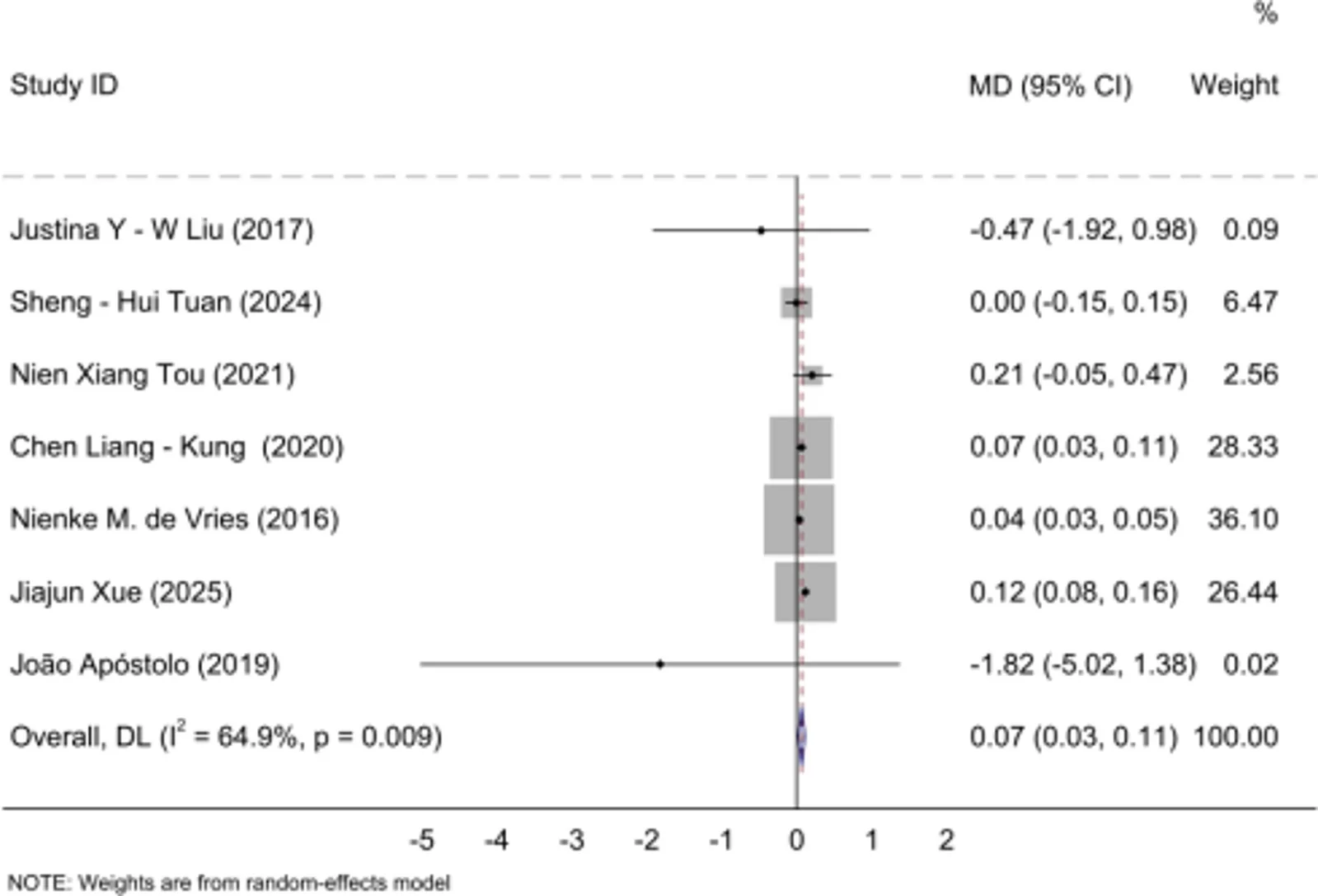
Forest plot of gait speed (m/s) analysis

A total of seven studies were included in the analysis of gait speed [16, 19, 22, 24–27]. Gait speed was assessed using either the Short Physical Performance Battery gait speed subscale or stopwatch-based timed walk tests over 4–10 m at habitual walking speed, with results reported in metres per second. The heterogeneity test indicated substantial heterogeneity among studies (I² = 54.9%, *P* = 0.009). The results showed that post-intervention gait speed was significantly higher in the nudging intervention group compared with the control group [MD = 0.07, 95% CI (0.03, 0.11), *P* = 0.001] (Fig. 7).

### Timed Up and Go test

**Fig. 8:**
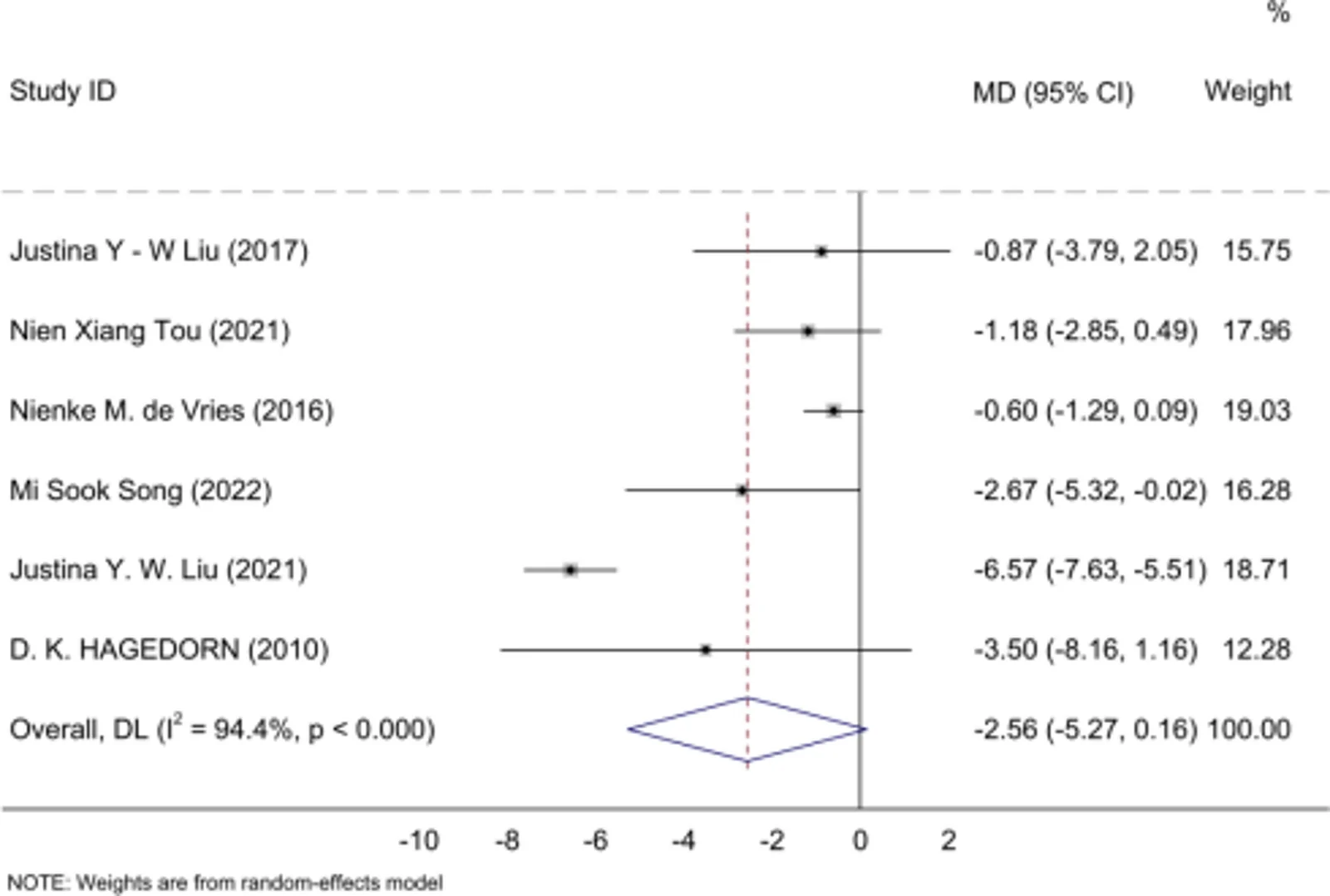
Forest plot of Timed Up and Go test (s) analysis

A total of six studies were included in the meta-analysis evaluating the effect of nudging-based physical activity interventions on the Timed Up and Go (TUG) test (in seconds) [19, 24, 27–30]. The TUG test was performed by measuring the time (in seconds) required for participants to rise from a seated position, walk three metres, turn, return to the chair, and sit down. The heterogeneity test indicated considerable heterogeneity among studies (I² = 94.4%, *P* < 0.001). The results showed no statistically significant between-group difference in post-intervention TUG time [MD = − 2.56, 95% CI (–5.27, 0.16), *P* = 0.065] (Fig. 8).

### Psychological and cognitive function–related indicators

#### Depressive symptoms

**Fig. 9:**
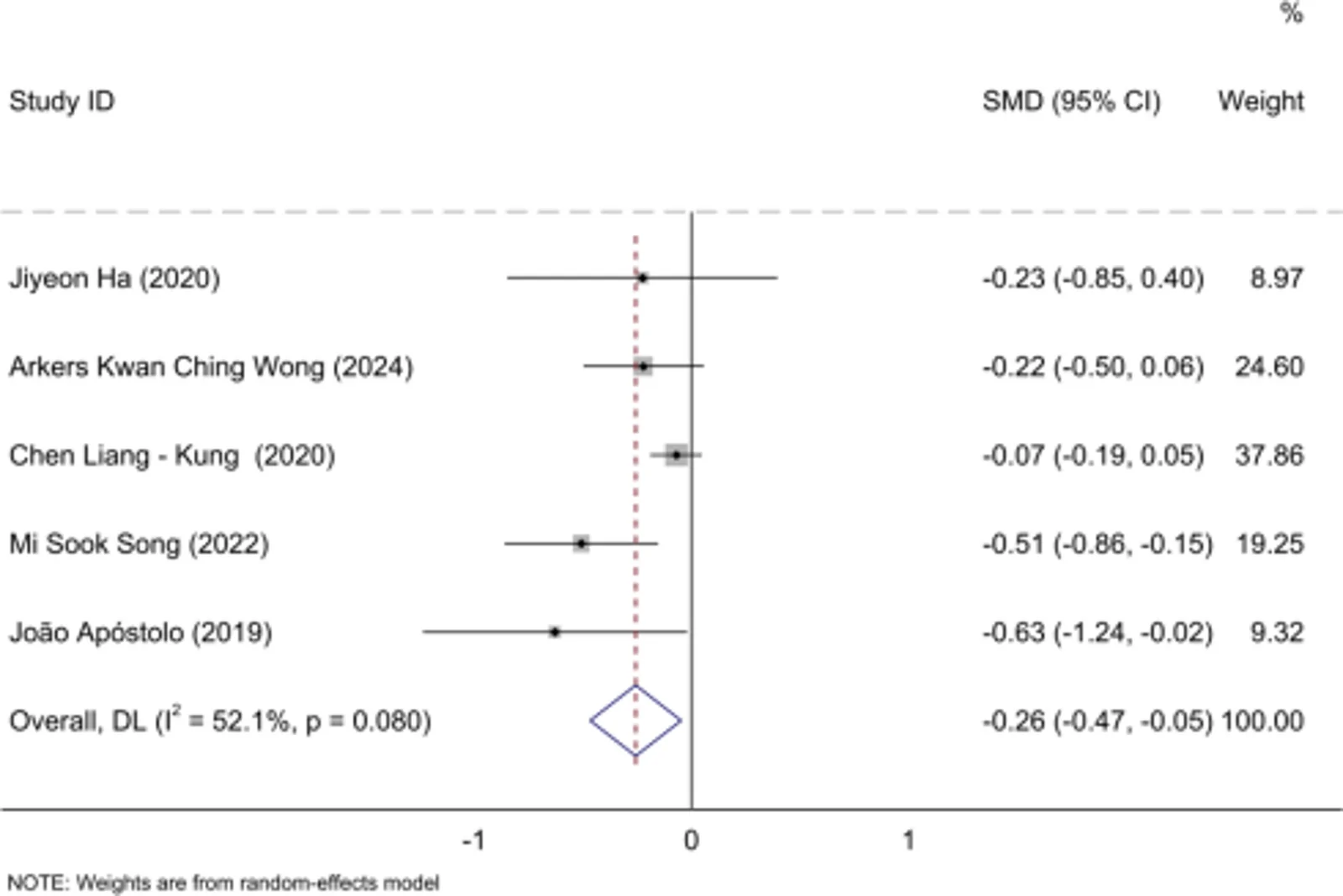
Forest plot of depressive symptom analysis

Five studies [15, 20, 25–26, 28] reported the effects of nudging-based physical activity interventions on depressive symptoms among frail older adults. Depressive symptoms were assessed using validated short-form versions of the Geriatric Depression Scale (5-, 10-, or 15-item) with culturally adapted, language-specific editions (e.g., Korean, Chinese, Portuguese), employing standard cutoff scores to classify symptom severity. Tests for heterogeneity indicated the substantial heterogeneity among the included studies (I² = 53.3%, *P* = 0.057). The results showed that post-intervention depressive symptom scores were significantly lower in the intervention group compared with the control group [SMD = − 0.30, 95% CI (–0.51, − 0.09), *P* = 0.016] (Fig. 9).

### Physical activity self-efficacy

**Fig. 10:**
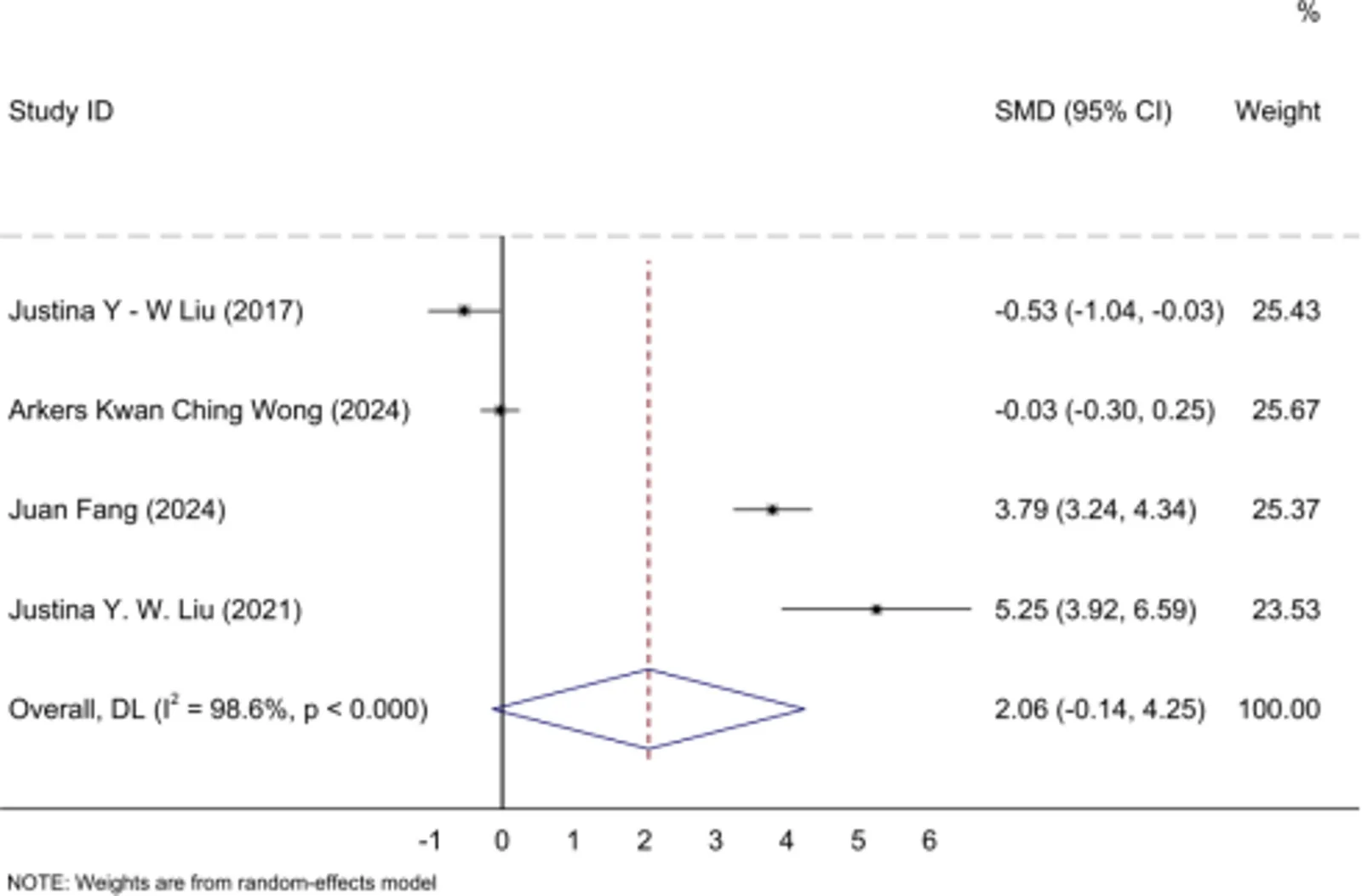
Forest plot of physical activity self-efficacy analysis

Four studies [19–21, 29] were included in the analysis of physical activity–related self-efficacy among frail older adults. Self-efficacy was measured using validated Chinese versions of self-report Likert scales, including the 9-item Self-Efficacy for Exercise Scale in three studies and the 10-item General Self-Efficacy Scale in one study. The heterogeneity test indicated extremely high heterogeneity among studies (I² = 98.6%, *P* < 0.001). The meta-analysis results showed that post-intervention self-efficacy scores were slightly higher in the nudging intervention group compared with the control group, although the difference did not reach statistical significance [SMD = 2.06, 95% CI (–0.14, 4.25), *P* = 0.066] (Fig. 10).

#### Cognitive function

**Fig. 11:**
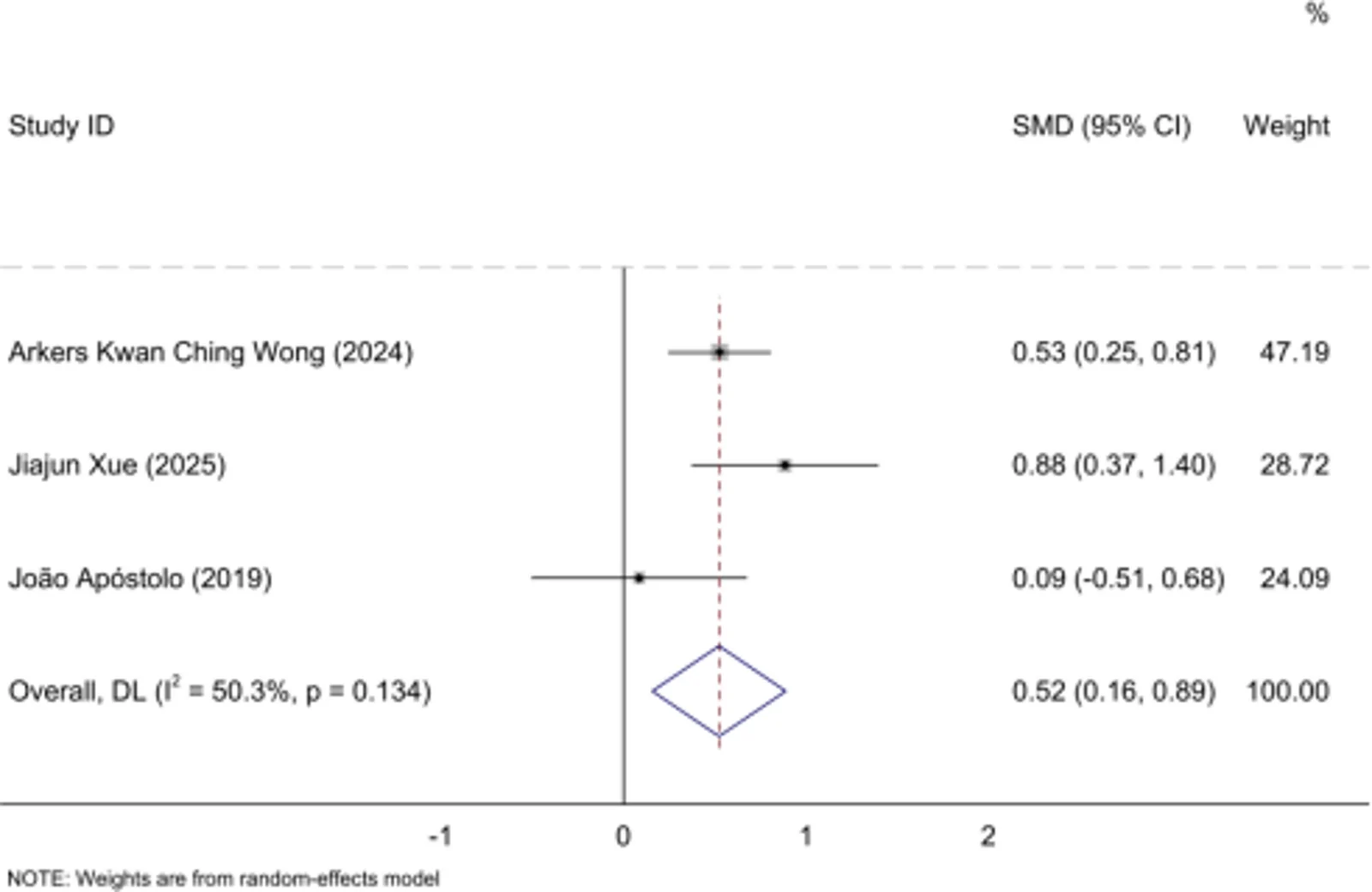
Forest plot of cognitive function analysis

A total of three studies [16, 20, 26] were included in the analysis of the effects of nudging-based physical activity interventions on cognitive function among frail older adults. Cognitive function was assessed using culturally adapted versions of the Montreal Cognitive Assessment (MoCA)—including the Peking, Hong Kong, and Portuguese editions. The heterogeneity test indicated moderate heterogeneity across studies (I² = 49.4%, *P* = 0.115). The results showed that post-intervention cognitive function scores were significantly higher in the intervention group compared with the control group [SMD = 0.47, 95% CI (0.26, 0.69), *P* = 0.005] (Fig. 11).

### Quality of life

**Fig. 12:**
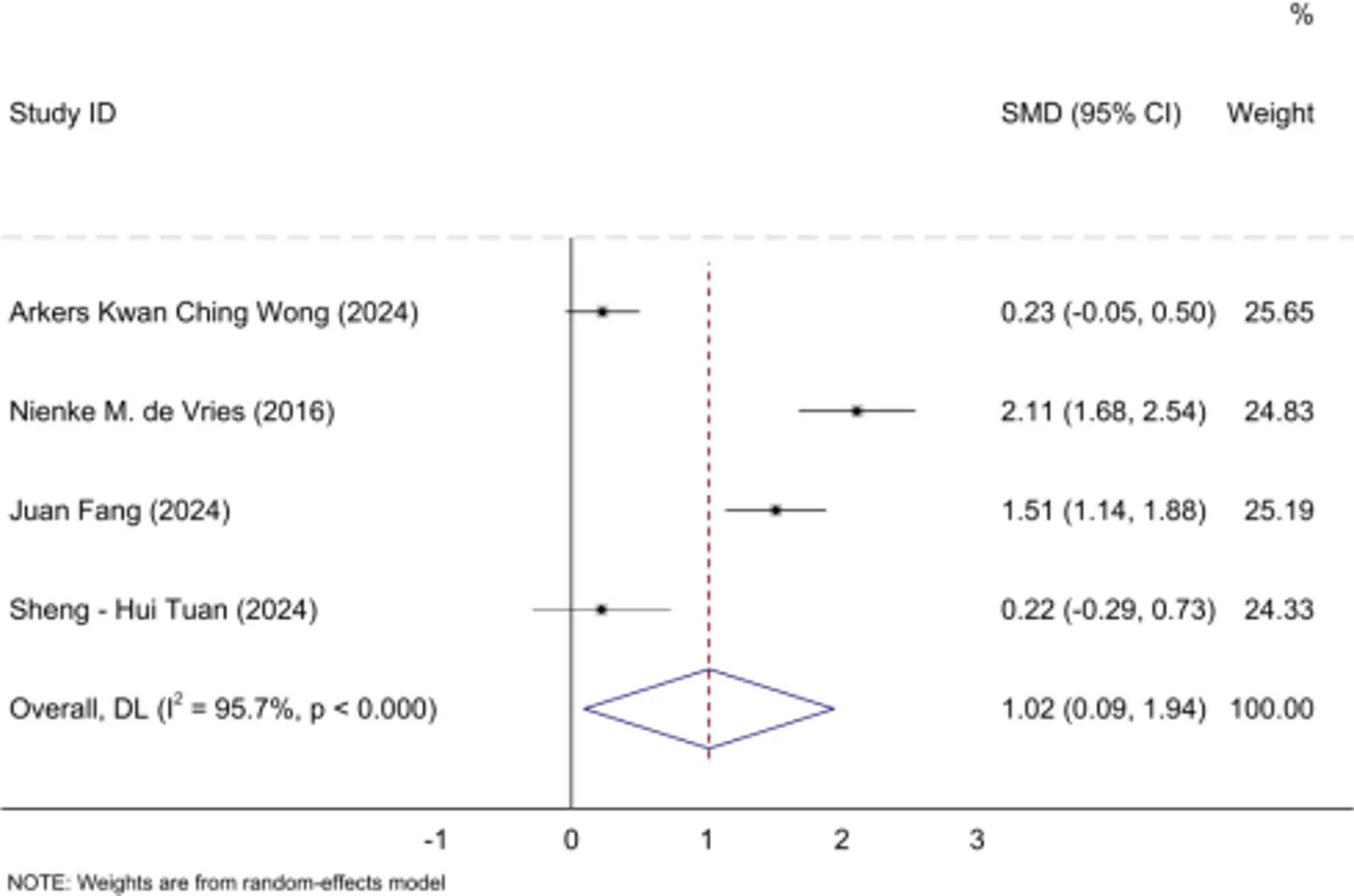
Forest plot of quality of life analysis

A total of four studies [17, 20, 26–27] were included in the analysis of QoL. QoL was assessed using the 12-Item Short-Form Health Survey, version 2 (SF-12v2) and 36-Item Short-Form Health Survey (SF-36) in two studies, with two studies further evaluating functional capacity as a proxy indicator of QoL using the Barthel Index and Lawton and Brody Scale; all measures were standardized to facilitate pooled analysis, with higher scores indicating better perceived health status and functional independence. The heterogeneity test revealed considerable heterogeneity among studies (I² = 95.7%, *P* < 0.001). The meta-analysis results indicated that post-intervention QoL was significantly better in the nudging-based intervention group than in the control group [SMD = 1.02, 95% CI (0.09, 1.94), *P* = 0.031] (Fig. 12).

### Sensitivity analysis

Leave-one-out sensitivity analyses were performed for all outcomes (Supplementary file 2, Figure S1-S10). The direction and statistical significance of the pooled effects were generally preserved for physical activity, frailty status, gait speed, and cognitive function, and the non-significant conclusion was unchanged for balance ability and physical activity self-efficacy. For grip strength, depressive symptoms, QoL, and TUG performance, the pooled effects showed greater sensitivity to individual study exclusion, consistent with the small study numbers and observed heterogeneity in these analyses.

### Publication bias

To systematically evaluate potential publication bias in this study, funnel plots, Begg’s rank correlation test, and Egger’s linear regression test were applied to all outcome indicators. First, the funnel plots (Supplementary file 3, Figure S11-S20) showed that the effect sizes for most outcomes were approximately symmetrically distributed, suggesting a generally balanced distribution of study effects. However, the funnel plots for “physical activity level” and “frailty score” appeared asymmetric, indicating potential publication bias. Further assessment using Begg’s and Egger’s tests (Table 2) revealed that in Begg’s test, the continuity-corrected Z values for most indicators were not statistically significant (*P* > 0.05), showing no evidence of publication bias. In contrast, Egger’s test indicated potential publication bias in the meta-analyses of “physical activity level” and “frailty score” (*P* < 0.05). However, these visual and inferential assessments should be interpreted with caution: visual inspection of funnel plots is unreliable with small numbers of studies, and Begg’s and Egger’s tests are poorly powered to detect publication bias when few studies are included; consequently, asymmetry may not reflect genuine bias, and nonsignificant results do not rule out the presence of bias.

**Table 2:** Tests for publication bias

Indicator	Begg’s		Egger’s	
	Z	*P*	bias	*P*
Balance ability	-0.34	1	0.49	0.345
Depressive symptoms	0.24	0.806	-1.91	0.075
Physical activity level	1.88	0.06	6.87	0.01
Quality of life	0.34	0.734	8.22	0.557
Physical activity self-efficacy	1.02	0.308	11.1	0.32
Frailty score	1.36	0.175	-7.42	0.039
Cognitive function	0	1	-0.53	0.905
Grip strength	0	1	3.71	0.109
Gait speed	0.6	0.548	0.47	0.6
Timed Up and Go test	0.75	0.452	-1.18	0.751
Overall	0.39	0.694	0.25	0.448

## Discussion

This review provides the first comprehensive synthesis of evidence on nudge-based physical activity interventions specifically targeting frail older adults. Drawing on 16 randomized controlled trials (RCTs), the findings reveal a differentiated pattern of effects: robust and statistically significant benefits were observed for global measures of physical activity, frailty severity, gait speed, depressive symptoms, cognitive performance, and QoL, whereas outcomes requiring greater neuromuscular precision—including upper limb strength, balance, and complex functional mobility—demonstrated directional improvement without attaining conventional statistical significance. Physical activity self-efficacy also failed to reach significance, likely reflecting measurement heterogeneity and variability in nudge operationalization across studies. These findings align with the overarching aim of evaluating whether subtle modifications to choice architecture, when embedded within multicomponent physical activity programs, can yield clinically meaningful health gains across the multidimensional frailty spectrum. The subsequent sections discuss each outcome domain in detail, with explicit delineation of nudge and non-nudge intervention components to facilitate accurate interpretation.

### Application effect of nudge strategy interventions on physical activity in frail older adults

It is important to note that the interventions included in this review were multicomponent in nature: they combined nudge strategies with non-nudge components. In the examples discussed below, we explicitly distinguish nudge components (e.g., reminders, feedback, social norm messages, gamification cues) from non-nudge components (e.g., structured training, professional coaching, educational sessions) to facilitate accurate interpretation of the findings. The results of this study indicate that nudge strategies were associated with significantly higher post-intervention physical activity levels among frail older adults. Despite the high heterogeneity, the conclusion that nudge-based interventions improve physical activity levels remains robust. Frail older adults often experience difficulties maintaining regular physical activity due to declines in cognitive function, lack of motivation, or environmental barriers, which impede rational decision-making [31]. Nudge strategies, which guide individuals toward healthier choices without restricting their freedom of choice, act on the intuitive decision-making system and reduce the psychological resistance to initiating behavior [32]. By employing non-coercive approaches such as information feedback, social incentives, and behavioral reminders, these strategies effectively overcome behavioral inertia and enhance adherence to physical activity among frail older adults. For instance, a large-scale community intervention conducted in Taiwan by Chen et al. [25] demonstrated that a comprehensive nudge-based program combining pedometer distribution, group competitions, and leadership by community role models significantly increased the daily step count of frail older adults. Similarly, Xue et al. [16] implemented an intervention in mainland China that integrated exercise video appliaction notifications, graded training, and peer support, resulting in nearly a 40-minute increase in weekly moderate-to-vigorous physical activity among frail older adults. In summary, nudge strategies—characterized by their low cost and minimally intrusive nature—show strong feasibility and effectiveness in promoting physical activity among frail older adults. They represent a promising behavioral intervention approach that warrants broader application and further promotion in future community and clinical health programs.

### Application effects of nudge-based physical activity interventions on psychological and cognitive functions in frail older adults

#### Depressive symptoms

This study found that nudge-based physical activity interventions were associated with significantly lower post-intervention depressive symptom scores among frail older adults. The sensitivity analysis indicated that these findings were not robust, suggesting that the pooled estimate may be sensitive to individual study characteristics and should be interpreted with caution. With advancing age and declining physiological reserves, frail older adults are more prone to mood disorders. Physical activity, as a non-pharmacological intervention, has been shown to exert antidepressant effects by modulating the hypothalamic–pituitary–adrenal (HPA) axis and promoting the release of endogenous opioid peptides [33–34]. Nudge strategies reduce the cognitive load required to initiate behavior, thereby enhancing frail older adults’ perceived control over healthy behaviors. In addition, key nudge elements—such as social support, positive feedback, and group interaction—help strengthen social connections, mitigate loneliness, and reduce psychological isolation, thus indirectly improving mood [11, 35]. For example, in the study by Liu et al. [19], interventions involving peer experience sharing and goal visualization significantly increased the emotional engagement of frail older adults in physical activity, demonstrating the psychological benefits of social support–based nudging strategies. Therefore, the positive psychological effects of nudge interventions offer a novel non-pharmacological approach for managing depression in older adults. Future research should further investigate the specific impacts of different types of nudges on emotional well-being among frail older adults.

#### Cognitive function

Meta-analysis results indicated that nudge-based physical activity interventions exerted a moderate positive effect on cognitive function in frail older adults. Sensitivity analysis demonstrated good robustness of the overall effect size, indicates that the conclusion is reliable. Moreover, after excluding the study by Xue et al. [16], which had the largest effect size, the pooled effect size slightly decreased, but the heterogeneity was markedly reduced, with the I² value dropping from 49.4% to approximately 30%. This suggests that this study may be the primary source of heterogeneity. Regular physical activity has been shown to increase hippocampal volume and improve executive function and working memory, mechanisms that involve upregulation of brain-derived neurotrophic factor (BDNF), enhanced cerebral blood flow, and reduction of neuroinflammation [36]. Several intervention programs included in this study integrated cognitive–motor training elements. For example, Apóstolo et al. [26] incorporated animal-assisted and group-based interactive seesions, which, while representing structured training elements rather than nudge strategies per se, were complemented by choice architecture techniques—reminders that facilitated participation. Suggesting that multicomponent interventions may exert synergistic neuroprotective effects. In this process, nudge strategies simplify decision-making procedures—such as presetting exercise times, providing real-time feedback, or introducing challenge-based tasks—thereby effectively addressing common issues in older adults like motivational delay and decline in executive functioning [7].

#### Physical activity self-efficacy

Although an overall upward trend in self-efficacy was observed, the pooled effect was not statistically significant, with high heterogeneity. The sensitivity analysis confirmed that non-significant conclusion was robust, suggesting that the non-significant result is unlikely to be driven by any single study. Nevertheless, the considerable heterogeneity indicates substantial inconsistency among studies, which may stem from three primary factors: (1) Differences in measurement tools across studies; (2) Inconsistent operational definitions of nudge types, with some studies emphasizing external incentives such as rewards, while others focused on intrinsic motivation strategies like self-goal setting; (3) Variations in baseline participant characteristics, especially in the level of retained cognitive function, which may have influenced individuals’ ability to process and internalize complex feedback information [37]. Therefore, future research should aim to standardize measurement tools for key constructs and further explore the mechanistic pathways through which different types of nudge strategies affect self-efficacy among frail older adults.

### Application effects of nudge-based physical activity interventions on frailty and physical function in frail older adults

#### Frailty status

This study found that physical activity interventions incorporating nudge strategies were associated with significantly lower post-intervention frailty scores among frail older adults, Sensitivity analysis demonstrated that the overall effect size was relatively robust, suggest that the overall conclusion regarding the intervention’s effect on frailty scores is reliable. Frailty, characterized by multi-system physiological decline, can be delayed or even reversed through non-pharmacological interventions [3]. Physical activity, as a core intervention, has been shown to mitigate the progression of frailty by improving muscle strength, energy metabolism, and neuroendocrine regulation [34]. Building on this, nudge strategies further enhance intervention effectiveness by optimizing choice architecture, helping older adults overcome motivational deficits and executive dysfunction, and promoting sustained participation in physical activity. For instance, Luger et al. [18] conducted a home-based intervention in Austria combining goal setting and family-based social support as core nudge strategies, alongside personalized health education. While goal setting functioned as a commitment device and family support leveraged social norms within the choice architecture, which effectively increased daily physical activity and significantly reduced frailty scores among older adults. Similarly, Romera-Liebana et al. [23] implemented a multifactorial intervention in primary care settings. While this program incorporated non-nudge components—including professional guidance, health education, and multicomponent exercise design—the behavioral drivers were embedded nudge strategies, namely group-based activities leveraging social approval, peer ambience, and default participation, coupled with dynamic intensity adjustments delivered through real-time feedback and automated adaptation. These choice architecture elements simplified decision-making and effectively enhanced exercise adherence while improving frailty outcomes among older adults.

#### Physical function indicators

In terms of functional outcomes, this study observed that nudge-based physical activity interventions were associated with significantly higher post-intervention gait speed among frail older adults. Sensitivity analysis indicated that even in the presence of substantial heterogeneity, the conclusion regarding the intervention effect on gait speed remained robust. Slower gait speed is a core indicator of frailty and an important predictor of falls and disability [1]. The findings suggest that even without mandatory high-intensity resistance training, nudge strategies—such as environmental cues, gamification, and personalized reminders—can effectively encourage walking behavior and improve walking efficiency. For instance, Tuan et al. [22] used the Ring Fit Adventure exergame for home-based exercise, where sensory feedback and adaptive difficulty adjustments significantly improved participants’ daily walking speed, highlighting the advantages of nudge-based interventions. However, the meta-analysis found no statistically significant improvement in the TUG test, and sensitivity analysis indicated that the results were heavily influenced by individual studies, with limited stability—thus requiring cautious interpretation. The TUG test reflects integrated motor abilities, encompassing sit-to-stand transitions, balance control, and gait planning [38]. Although nudge strategies may promote better motor performance by fostering consistent movement habits and reducing hesitation and fear [11, 39–40], the current evidence remains insufficient to confirm a definitive improvement in TUG performance, warranting further investigation. Similarly, although grip strength showed a slight upward trend, the effect was not statistically significant, with substantial heterogeneity. The sensitivity of the grip strength results to individual study exclusion is consistent with the high observed heterogeneity, suggesting that the non-significant finding may be attributable to between-study heterogeneity and differences in sample size. Grip strength mainly reflects upper-limb muscle power, which typically requires systematic resistance training for meaningful improvement [41–42]. Most existing nudge-based interventions target general physical activities—such as walking or flexibility exercises—without providing specific guidance or progression monitoring for muscle-strength training, which may explain the limited effects observed. Additionally, grip strength measurements are easily affected by factors such as testing posture, device model, and evaluator technique, potentially amplifying inter-study variability [43]. Likewise, improvements in balance ability were small and not statistically significant, the sensitivity analysis confirmed that the balance ability findings were robust. suggesting that nudge-based physical activity interventions alone cannot replace specialized vestibular–proprioceptive integration training.

#### Nudge-based physical activity interventions improve quality of life in frail older adults

The analysis results showed that frail older adults who received nudge-based physical activity interventions had significantly better post-intervention QoL than the control group. Notably, half of the included studies measured functional status using the Barthel Index (basic Activities of Daily Living [ADL]) and the Lawton and Brody Scale (Instrumental Activities of Daily Living [IADL]) rather than dedicated Health-Related Quality of Life (HRQoL) instruments; this inclusive approach was adopted because only two of the sixteen included studies reported dedicated HRQoL outcomes—a scarcity characteristic of this emerging intervention field. Functional independence is a core dimension of QoL in frail older adults, and ADL/IADL measures are well-validated and frequently used as QoL surrogates in geriatric research when self-reported HRQoL data are limited. All four studies demonstrated effects in the same favorable direction, supporting the interpretation of the pooled estimate as reflecting overall health-related well-being. However, Leave-one-out sensitivity analyses suggested that the pooled estimates were sensitive to individual study exclusion, with the omission of either Fang et al. [21] or Wong et al. [20]rendering the effect non-significant; these results should therefore be interpreted with caution. QoL is a multidimensional construct encompassing physical function, psychological well-being, social relationships, and environmental adaptation. Nudge strategies positively influence QoL by enhancing physical activity participation, improving self-management ability, and rebuilding social connections [44]. For example, de Vries et al. [27] found that a patient-centered physical therapy program incorporating nudge element—specifically, goal-setting feedback and family support cues, continuously improved the QoL of frail older adults over 24 weeks. Overall, by subtly guiding decision-making processes without coercion, nudge strategies facilitate improvements from physiological function to psychosocial adaptation, offering a feasible and effective pathway to enhance overall QoL in frail older adults. Therefore, it is recommended to integrate nudge mechanisms into chronic disease management and comprehensive geriatric intervention systems to ensure the sustainability of health promotion.

### Strengths and limitations

#### Strengths

To our knowledge, this is the first systematic review and meta-analysis to evaluate nudge-based physical activity interventions specifically targeting frail older adults. The review was prospectively registered and conducted in accordance with PRISMA guidelines, with systematic searches across nine electronic databases. The rigorous methodology included dual independent screening and data extraction with third-party arbitration, Cochrane risk-of-bias assessment, and comprehensive evaluation of outcomes spanning physical, psychological, cognitive, and functional domains. The inclusion of 16 RCTs comprising 2,730 participants provides a substantial evidence base. Our theoretically grounded operational definition of nudge - based interventions enhances transparency and reproducibility.

#### Limitations

Several limitations should be acknowledged. First, the methodological quality varied across studies: 18.8% and 25% of studies were at high risk-of-bias for random sequence generation and allocation concealment, respectively, and approximately 50% were at unclear or high risk for blinding of participants and personnel. Although blinding is inherently challenging in behavioural interventions, inadequate blinding may have inflated effect estimates for subjective outcomes. Second, substantial statistical heterogeneity was observed across most outcomes, reflecting diversity in intervention types, settings, and nudge strategies. Due to the limited number of studies for each outcome, formal subgroup analyses or meta-regression were not feasible; Pooled estimates should be interpreted cautiously as average effects across heterogeneous contexts. Third, all interventions were multicomponent, combining nudge strategies with non-nudge elements (e.g., health education, professional guidance), precluding isolation of the independent effect of nudge components. Fourth, Egger’s test suggested potential publication bias for physical activity level and frailty score, though these assessments are underpowered with few studies. Trim-and-fill analyses were not performed because this method has been shown to yield unreliable imputations when applied to meta-analyses with few studies; future reviews with a larger evidence base should consider incorporating gray literature searches and trim-and-fill sensitivity analyses to further assess and control for potential publication bias. Sensitivity analyses indicated that pooled effects for depressive symptoms, QoL, TUG, and grip strength were sensitive to exclusion of individual studies, limiting the reliability of these findings. Fifth, the QoL analysis was constrained by limited availability of dedicated HRQoL data—only two studies employed the SF-12v2 or SF-36—necessitating inclusion of functional status measures (Barthel Index, Lawton and Brody Scale) as proxy indicators. Although functional independence is strongly correlated with QoL in this population, this construct heterogeneity limits interpretability of the pooled estimate as a pure measure of HRQoL. Finally, most interventions were of short duration (12–24 weeks) with limited long-term follow-up, precluding conclusions about sustainability of effects.

## Conclusions

This systematic review demonstrates that nudge-based physical activity interventions can significantly increase physical activity levels, improve frailty status, depressive symptoms, cognitive function, gait speed, and QoL in frail older adults. Although improvements in self-efficacy, grip strength, balance ability, and TUG performance were not statistically significant—possibly due to insufficient intervention intensity, small sample sizes in individual trials, or measurement heterogeneity—the overall evidence indicates that these interventions offer low-cost, highly feasible strategies to delay the progression of frailty and are well-suited for community and home-based implementation. Future studies should aim to standardize the classification and measurement of nudge strategies, elucidate the comparative effectiveness of different nudge types across specific outcomes, and evaluate long-term maintenance of intervention benefits through adequately powered RCTs with extended follow-up. Embedding rigorously designed nudge components within existing community and primary care delivery systems represents a promising, scalable approach to promoting sustained physical activity engagement and attenuating frailty progression in aging populations.

## Supplementary Information

Supplementary Material 1.

Supplementary Material 2.

Supplementary Material 3.

Supplementary Material 4.

## Data Availability

All data pertinent to this study are available within the article. For any additional information, please contact the corresponding author.

## Abbreviations

SMD: Standardized Mean Difference
MD: Mean Difference
PRISMA: Preferred Reporting Items for Systematic Reviews and Meta-analyses
TUG: Timed Up and Go
HPA: Hypothalamic–Pituitary–Adrenal
BDNF: Brain-derived Neurotrophic Factor
CI: Confidence Interval
PICO: Population, Intervention, Comparison, Outcome
MeSH: Medical Subject Headings
SD: Standard Deviation
CKNI: China National Knowledge Infrastructure
EMBASE: Excerpta Medica Database
PsycINFO: Psychological Information
EconLit: Economic Literature
SF-12v2: 12-Item Short-Form Health Survey, version 2
SF-36: 36-Item Short-Form Health Survey
DL: DerSimonian-Laird
SHARE-FI: Survey of Health, Ageing and Retirement in Europe Frailty Instrument
MoCA: Montreal Cognitive Assessment
HRQoL: Health-Related Quality of Life
QoL: Quality of Life
ADL: Activities of Daily Living
IADL: Instrumental Activities of Daily Living
RCTs: Randomized Controlled Trials
