# Supplementary material for: The effectiveness of nudge-based physical activity interventions in frail older adults: a systematic review
Source: Int J Behav Nutr Phys Act. 2026 Jul 7;23:90. doi: 10.1186/s12966-026-01948-3 (PMC13632310; doi:10.1186/s12966-026-01948-3)
Supplement: Supplementary file 2 [file 12966_2026_1948_MOESM1_ESM.docx]

**Supplementary file 2**

**Supplementary Table S1. Example of Search Strategy in PubMed.**

| **Step** | **Search Strategy** |
| --- | --- |
| #1 | ("elder*" OR "senior*" OR "old adults*" OR "older people" OR "older population" OR "Aged" OR "geriatric*" OR "aging" OR "retire*" OR "Aged"[Mesh] OR "Frail Elderly"[Mesh])[Title/Abstract] |
| #2 | ("frail*" OR "weak" OR "frailty" OR "fragile" OR "fragility" OR "debility" OR "weakness" OR "frailty syndrome" OR "asthenia" OR "hypothenia" OR "frailness" OR "Frailty"[Mesh])[Title/Abstract] |
| #3 | ("Exercise" OR "exercise*" OR "physical activit*" OR "sport*" OR "physical fitness" OR "training*" OR "Exercise"[Mesh] OR "Sports"[Mesh] OR "Physical Fitness"[Mesh])[Title/Abstract] |
| #4 | ("nudg*" OR "choice architect*" OR "paternalis*" OR "behavioral economics" OR "behavioural economics")[Title/Abstract] |
| #5 | ("decision structur*" OR "decision information" OR "decision assist*" OR "incentivi*" OR "norm" OR "norms" OR "social norm" OR "social proof*" OR "messenger" OR "opinion leader" OR "remind*" OR "commit*" OR "pre-commitment" OR "feedback*" OR "peer support*" OR "peer group*" OR "peer comparison*" OR "social support*" OR "social influen*" OR "default*" OR "salienc*" OR "affect*" OR "ego" OR "alert*" OR "fram*" OR "loss frame" OR "gain frame" OR "encourag*" OR "motivat*" OR "persua*" OR "persuasive communication*" OR "risk communication*" OR "health communication*" OR "heuristic*" OR "cue" OR "cues" OR "prompt*" OR "prime" OR "priming" OR "simplif*" OR "disclosure" OR "warning*" OR "graphic*" OR "poster*" OR "goal setting")[Title/Abstract] |
| #6 | (randomized controlled trial[Publication Type] OR controlled clinical trial[Publication Type] OR randomized[Title/Abstract] OR placebo[Title/Abstract] OR randomly[Title/Abstract] OR trial[Title/Abstract] OR groups[Title/Abstract]) NOT (animals[Mesh] NOT humans[Mesh]) |
| #7 | #1AND #2AND#3AND(#4OR#5)AND#6 |
