# Supplementary material for: The effectiveness of nudge-based physical activity interventions in frail older adults: a systematic review
Source: Int J Behav Nutr Phys Act. 2026 Jul 7;23:90. doi: 10.1186/s12966-026-01948-3 (PMC13632310; doi:10.1186/s12966-026-01948-3)
Supplement: Supplementary file 3 [file 12966_2026_1948_MOESM2_ESM.docx]

**Supplementary file 2**

Supplementary Figure S1. Sensitivity analysis of physical activity outcomes.

Supplementary Figure S2. Sensitivity analysis of frailty score.

Supplementary Figure S3. Sensitivity analysis of balance ability.

Supplementary Figure S4. Sensitivity analysis of grip strength (kg).

Supplementary Figure S5. Sensitivity analysis of gait speed (m/s).

Supplementary Figure S6. Sensitivity analysis of Timed Up and Go test (s).

Supplementary Figure S7. Sensitivity analysis of depressive symptoms.

Supplementary Figure S8. Sensitivity analysis of physical activity self-efficacy.

Supplementary Figure S9. Sensitivity analysis of cognitive function.

Supplementary Figure S10. Sensitivity analysis of quality of life.

**Figure S1. Sensitivity analysis of physical activity outcomes.**

**Figure S2. Sensitivity analysis of frailty score.**

**Figure S3. Sensitivity analysis of balance ability.**

**Figure S4. Sensitivity analysis of grip strength (kg).**

**Figure S5. Sensitivity analysis of gait speed (m/s).**

**Figure S6. Sensitivity analysis of Timed Up and Go test (s).**

**Figure S7. Sensitivity analysis of depressive symptoms.**

**Figure S8. Sensitivity analysis of physical activity self-efficacy.**

**Figure S9. Sensitivity analysis of cognitive function.**

**Figure S10. Sensitivity analysis of quality of life.**
