# Supplementary material for: The effectiveness of nudge-based physical activity interventions in frail older adults: a systematic review
Source: Int J Behav Nutr Phys Act. 2026 Jul 7;23:90. doi: 10.1186/s12966-026-01948-3 (PMC13632310; doi:10.1186/s12966-026-01948-3)
Supplement: Supplementary file 4 [file 12966_2026_1948_MOESM3_ESM.docx]

**Supplementary file 3**

Supplementary Figure S11. Funnel plot of gait speed intervention effect.

Supplementary Figure S12. Funnel plot of physical activity intervention effect.

Supplementary Figure S13. Funnel plot of balance ability intervention effect.

Supplementary Figure S14. Funnel plot of Timed Up and Go test intervention effect.

Supplementary Figure S15. Funnel plot of cognitive function intervention effect.

Supplementary Figure S16. Funnel plot of quality of life intervention effect.

Supplementary Figure S17. Funnel plot of frailty score intervention effect.

Supplementary Figure S18. Funnel plot of grip strength intervention effect.

Supplementary Figure S19. Funnel plot of depressive symptoms intervention effect.

Supplementary Figure S20. Funnel plot of physical activity self-efficacy intervention effect.

**Figure S11. Funnel plot of gait speed intervention effect.**

**Figure S12. Funnel plot of physical activity intervention effect.**

**Figure S13. Funnel plot of balance ability intervention effect.**

**Figure S14. Funnel plot of Timed Up and Go test intervention effect.**

**Figure S15. Funnel plot of cognitive function intervention effect.**

**Figure S16. Funnel plot of quality of life intervention effect.**

**Figure S17. Funnel plot of frailty score intervention effect.**

**Figure S18. Funnel plot of grip strength intervention effect.**

**Figure S19. Funnel plot of depressive symptoms intervention effect.**

**Figure S20. Funnel plot of physical activity self-efficacy intervention effect.**
